# Temperature induced Spin Switching in SmFeO_3_ Single Crystal

**DOI:** 10.1038/srep05960

**Published:** 2014-08-05

**Authors:** Shixun Cao, Huazhi Zhao, Baojuan Kang, Jincang Zhang, Wei Ren

**Affiliations:** 1 Department of Physics, Shanghai University, 99 Shangda Road, Shanghai 200444, China

## Abstract

The prospect of controlling the magnetization (*M*) of a material is of great importance from the viewpoints of fundamental physics and future applications of emerging spintronics. A class of rare-earth orthoferrites RFeO_3_ (R is rare-earth element) materials exhibit striking physical properties of spin switching and magnetization reversal induced by temperature and/or applied magnetic field. Furthermore, due to the novel magnetic, magneto-optic and multiferroic properties etc., RFeO_3_ materials are attracting more and more interests in recent years. We have prepared and investigated a prototype of RFeO_3_ materials, namely SmFeO_3_ single-crystal. And we report magnetic measurements upon both field cooling (FC) and zero-field cooling (ZFC) of the sample, as a function of temperature and applied magnetic field. The central findings of this study include that the magnetization of single-crystal SmFeO_3_ can be switched by temperature, and tuning the magnitude of applied magnetic field allows us to realize such spin switching even at room temperature.

The emergent spintronics calls for novel materials with unprecedented controls of magnetism^1^,^2^,^3^,^4^. Rare-earth orthoferrites RFeO_3_ (R is rare-earth element) materials are excellent examples to show temperature and/or magnetic field induced spin switching and magnetization reversal^1^,^4^,^5^,^6^,^7^, intriguing magnetic^8^,^9^,^10^, magneto-optic^4^,^11^,^12^ and multiferroic^13^,^14^,^15^ properties. RFeO_3_ possesses two magnetic sublattices, from 4f-electrons of the rare-earth ions (R-sublattice) and 3d-electrons of the iron ions (Fe-sublattice), respectively. At relatively higher temperatures, a weak ferromagnetism (FM) originates from the canted antiferromagnetism (AFM) in the Fe-sublattice^16^,^17^, while the R-sublattice only orders antiferromagnetically at much lower temperatures. Moreover, there exists a delicate exchange interplay between R 4f- and Fe 3d-electrons in these complex oxides. As a result, interesting magnetic behaviors are strongly dependent on several external stimulations, such as applied magnetic field, temperature, and pressure etc.

Among many family members of RFeO_3_, SmFeO_3_ has been shown to have excellent device characteristics such as the fast magnetic switching^7^, and an easy axis rotation transition (also known as spin reorientation transition) from *c*-axis to *a*-axis which occurs at quite high temperature between *T_SR1_* = 450 K and *T_SR2_* = 480 K^14^,^18^ (see Figure 1). This is the highest spin reorientation transition temperature of the whole RFeO_3_ family, which indeed deserves special attention for practical uses. At the same time, the interplay between Sm 4f- and Fe 3d-electrons is also intriguing^14^,^18^,^19^,^20^. For example, below 4 K, SmFeO_3_ exhibits an interesting phenomenon of spontaneous magnetization reversal. This reversal is attributed to the activation of the long range ordering of Sm^3+^ spins whose total magnetic moment is antiparallel to the weak ferromagnetic moment of canted AFM ordering of Fe-sublattice^14^. Moreover, the onset of strong competition interplay between Sm-4f and Fe-3d electrons can be observed to occur at a relatively high temperature of 140 K, below which the total magnetization is monotonically suppressed and dramatically reversed sign at low temperature^7^,^19^,^20^.

We first look at the notable feature of single crystal SmFeO_3_ well above room temperature. The magnetic anisotropy dependence of temperature can be obtained by applying external magnetic fields along *a*- and *c*-axes, i.e. *H*//*a* and *H*//*c* to measure magnetizations *M_a_* and *M_c_*, as illustrated in Figure 1. The magnitude of the applied magnetic field is *H* = 300 Oe. Below its Néel temperature *T_N_* = 680 K, SmFeO_3_ becomes a canted antiferromagnet with an FM vector from Fe-sublattice along *c*-axis as indicated by the red curve. The marked region in a dashed rectangle near 450–480 K exhibits the spin reorientation transition of the Fe-sublattice, where *M_a_-T* and *M_c_-T* show crossover behavior with exchanged magnetization magnitude. This continuous transition (so-called Γ_2_(*G_z_*, *F_x_*) to Γ_4_(*G_x_*, *F_z_*) magnetic configuration, i.e. canted *G*-type AFM and resultant weak *FM* along *a*, *c* directions) is in good agreement with that reported in Refs. [14,18,20] for SmFeO_3_ single crystal.

We further discover here, below a certain critical point near room temperature, *M_a_-T* curves after FC and ZFC may exhibit interesting magnetic behaviors of opposite signs (green and black curves in Figure 1). At such critical temperature (*T_ssw_* = 278.5 K for *H* = 300 Oe), we observe a very sharp magnetization jump that clearly demonstrates to a spin switching transition. For both FC and ZFC, a compensation temperature (*T_comp_* = 3.9 K for *H* = 300 Oe) corresponding to zero magnetization is obtained. Note that near *T_comp_*, *M_a_-T* dramatically decreases in magnitude and changes its sign, whereas *M_c_-T* curve remains essentially a zero magnetization below *T*_SR1_. In the FC case, *M_a_-T* shows a less temperature-dependent form in a large temperature range of 100–350 K, and two strongly temperature-sensitive regimes at the compensation and spin-reorientation temperatures. This measured behavior of *M_a_-T* after the FC is also in consistent with Refs. [7,14,20] when *H* = 100 or 500 Oe, but the more interesting *M_a_-T* curve measured after the ZFC has never been reported.

Let us now focus on the *M_a_-T* measured after the ZFC along *a*-axis of single-crystal SmFeO_3_ as shown in Figure 2, under an applied magnetic field of *H* = 300 Oe. When we increase the temperature from 3 K to 350 K, the total magnetization *M_a_* falls rapidly and changes sign by crossing a full annihilation (zero magnetization) at 3.9 K, and then achieves a relatively saturate *negative* magnetization above 100 K. Note that a similar compensation behavior was also reported in other RFeO_3_ (R = Nd, Er)^5^,^21^ and in RMnO_3_ (R = Nd, Sm)^22^,^23^,^24^ systems. As consistent with what is advocated in the literature, we interpret such feature as indicative that the resulting magnetization associated with the FM vector of Sm-sublattice considerably decreases in size (with respect to that of Fe-sublattice) when the temperature increases (see the red and blue arrows illustrating the relative directions and magnitudes of FM vectors of Sm- and Fe-sublattices). At *T_comp_* = 3.9 K, the FM vector of Sm-sublattice is equal in magnitude to that of Fe-sublattice but reversed in direction (AFM-type coupled with each other), producing in a vanishing total magnetization. For temperatures above *T_comp_* = 3.9 K and below *T_ssw_* = 278.5 K, the FM vector of Sm-sublattice becomes smaller than that of Fe-sublattice (and still reversed in direction), therefore explaining why the total net magnetization is *negative*. After a fast decrease, the total magnetization reaches its relative saturation in the temperature region of 100–278.5 K, since the magnetic ordering of the Sm-sublattice is weakened *by temperature* much faster than that of the Fe-sublattice.

Figure 2 also highlights the main finding of this work, namely a remarkable first-order transition at *T_ssw_* = 278.5 K. The magnetization undergoes a sudden jump with another sign change from *negative* to *positive* value, showing a mirror-like symmetry with respect to zero magnetization at transition point. We note that the observed switching with temperature is not reversible, since our ZFC curve is obtained in a warming up process. This distinctive spin switching effect should thus be associated with a spontaneous spin-flip transition of Sm- and Fe-sublattices at the same time, accompanied by an exchange of their FM vector directions. This is in analogy with what we observed in NdFeO_3_ near 29 K when *H* = 100 Oe^5^. However, the *negative* *M_a_* exists in a much wider temperature range from *T_comp_* = 3.9 K to near room temperature of *T_ssw_* = 278.5 K in SmFeO_3_ system, whereas in NdFeO_3_ it only happens in a narrow temperature region from 7.6 K to 29 K. This indicates that the unyielding feature of Sm-sublattice that keeps its magnetization parallel to the field *H* and antiparallel to magnetization of Fe-sublattice up to a high temperature (even at *T_ssw_* = 356 K for *H* = 250 Oe as shown below). This striking result reveals an evidence for the existence of strong interaction between Sm-4f and Fe-3d electrons in SmFeO_3_ system. And we speculate that the Sm-sublattice may have a long-range ordering to some extent even near room temperature.

In order to study the relationship between the spin switching temperature *T_ssw_* and the applied magnetic field *H* with a possible modulation, we have measured the *M_a_-T* curves (in ZFC regime) under different *H*. We find that *T_ssw_* can be readily controlled in a wide temperature range by varying the magnitude of the applied magnetic field, as shown in Figure 3. Thus, *T_ssw_* is observed to be very field-sensitive, but the magnitudes of the positive and negative *M_a_* around *T_ssw_* is less sensitive to the initial increase of field (Figure 3a, Figure 3b). As the applied magnetic field is strengthened from 250 Oe to 20000 Oe, *T_ssw_* changes dramatically from 356 K to 4 K (Figure 3c). Note that for a large *H* >2000 Oe, the switched *M* values from negative to positive will become nonsymmetrical and a smaller spin jump to positive *M* will be observed. It is clear that, under low magnetic field of 250–600 Oe, *T_ssw_* covers the most useful temperature regime from 356 K to about 20 K and possible spin switching or magnetic sensor devices can be easily designed. For higher fields of 700–1000 Oe, the *T_ssw_-H* curve undergoes a knee point transition. And for the further high field of *H*>2000 Oe, the *T_ssw_-H* curve starts to reach a saturation. When *H* is 10000 Oe or higher, the negative magnetization and *T_comp_* totally vanishes, whereas the residual spin switching transition *T_ssw_* can still be observed with a very small magnetization jump, and the total magnetization *M_a_* is aligned parallel to the direction of field *H*.

According to the previously reported work, the rare earth Sm ions in SmFeO_3_ system seem to establish long-range ordering below 140 K^7^,^19^,^20^, which is the highest temperature in the rare earth orthoferrites RFeO_3_ compounds. For other RFeO_3_ systems like R = Nd, Er, Ho, etc., their onset temperatures of long-range ordering of rare earth sublattices are all below 100 K. Our first-principles calculation study^10^ for NdFeO_3_ system indicates that the spin reorientation transition of Fe-sublattice can be ascribed to the exchange interaction between Nd-4f and Fe-3d electrons, which are mediated by O-2p state. As the temperature decreases, the superexchange angle of Fe-O-Fe gets larger, the Fe-O and Nd-O bonds become more covalent, and the exchange interactions become stronger. This study reveals that 4f-electrons of rare earth ions play the main role in triggering the spin reorientation transition of Fe-sublattice. The magnetic properties (magnetization) of RFeO_3_ below spin reorientation temperature should be dominated by the rare-earth sublattice, which is FM-coupled or AFM-coupled with the net FM vector of Fe-sublattice. For our case of SmFeO_3_ system, the delicate interactions among R-R, R-Fe, and Fe-Fe ions, and the role of rare earth Sm-sublattice are similar with other RFeO_3_ family members. Nonetheless, the uniquely high *T_SR_* window (450–480 K) well above room temperature makes SmFeO_3_ system an exceptional material with spin switching *T_ssw_* at work near room temperature. Above *T_ssw_*, for both ZFC and FC, the total magnetization stays parallel to the *a*-axis and slightly decreases with increasing temperature until the spin reorientation transition region of Fe-sublattice (Figure 1). This result suggests the gradual decrease of FM vector from Fe-sublattice and the progressive disappearance of the long-range ordering moments of Sm-sublattice, as consistent with literature of RFeO_3_ materials^18^.

Figure 4 shows the magnetic field dependence of the magnetization at the spin switching temperature of SmFeO_3_ single crystal, where the “lower” and “upper” data refer to as the magnitudes of magnetization before and after the spin switching transition, respectively. The green data in between are arithmetic average value of red and blue ones. When *H*<700 Oe, the spin switching behavior has a mirror symmetry between the *negative* and *positive* magnetizations, with their average being precisely equal to zero. When we apply an enhanced field of *H* = 700 Oe and higher, such average magnetization shifts to more *positive* values as *H* increases. Moreover, the magnetization jump Δ*M* (or the magnetization difference) before and after the spin switching transition increases and then decreases with increasing *H*, by showing a peak at *H* = 500–600 Oe. From Figure 3 and Figure 4, we verify that the spin switching effect is significantly suppressed by the applied field larger than 800 Oe, but is perfectly controllable at modestly low field. When *H* is higher than 1000 Oe, the transition temperature *T_ssw_* is suppressed to be below 10 K, and the magnetization jump Δ*M* becomes more and more inconspicuous. Thus our experiments reveal the properly applied magnetic field may result highly versatile magnetic state due to the Sm-4f and Fe-3d magnetic sublattices. This feature makes SmFeO_3_ an easily manipulated candidate for practical application of spin switching devices by using very low field. Furthermore, it is likely that the key role of rare earth Sm ions might be found in many other perovskite ABO_3_ compounds. Since the Sm-4f electrons may trigger novel effects of spin state transitions near room temperature, they may be applied in the future spintronics, quantum computations and quantum communications.

Figure 5 shows the magnetic field dependence of the compensation temperature *T_comp_* of SmFeO_3_ single crystal for the applied field *H* = 0–2000 Oe. Overall, *T_comp_* is relatively independent of the applied field, in a narrow range of 3.5 ~ 4.5 K. For larger field beyond 2000 Oe, the magnetization becomes always positive in the whole range of temperature. The slight rise of *T_comp_* with the increasing field is because that the magnetic field enhances the value of the positive magnetization at the lower temperatures. Note that interestingly *T_comp_-H* curve shows a small dip when *H* = 700–900 Oe, which is corresponding to the inflection point in the *T_ssw_-H* curve in Figure 3c and the sharp decrease of Δ*M* in Figure 4. These details again demonstrate that the strong exchange interaction between the Sm-4f and Fe-3d electrons renders SmFeO_3_ possessing extremely alterable spin configurations by small perturbations like weak magnetic field, especially near room temperature.

Let us now come back and pay more attention to the first-order transition occurring at *T*_ssw_, since such a sharp spin-reversal transition may be put in use for designing novel spin switching or magnetic sensor device. One may wonder what the origin of that first-order transition is. It is important to realize that, in addition to the exchange interaction between Sm-4f and Fe-3d electrons that result in opposite, temperature-dependent magnetizations in the Sm- and Fe-sublattices, the investigated rare-earth orthoferrite has also another energetic preference: it desires to have a total ferromagnetic moment being aligned along the field's direction. Such desire should become more and more pronounced, with respect to the interactions between Fe-3d and Sm-4f electrons, when increasing the magnitude of the applied field. Figure 3 indicates that this is indeed the case since enhancing the magnetic field from 250 to 2000 Oe significantly shifts *T_ssw_* towards lower temperature, therefore leading to a *maximum* *negative* magnetization that considerably reduces. In fact, for the field stronger than 2000 Oe, the interaction between the total magnetization and the applied field prevails over the intrinsic exchange interactions between Sm-4f electrons and Fe-3d electrons, since the magnetization is always positive for any studied temperature -- therefore annihilating the existence of a compensation temperature, but the temperature-induced spin flip is still observable through a very small jump (the so called spin switching effect in the above, see Figure 3). It is noticeable that, for the case of SmFeO_3_, the *T_ssw_* (under low field, such as 250 Oe) is much higher than that of other RFeO_3_ compounds (such as 29 K for NdFeO_3_, 65 K for ErFeO_3_ under 100 Oe), and the transition temperature can be modulated to a temperature as high as 356 K. This outstanding feature exhibits an evidence of strong exchange interaction between Sm-4f and Fe-3d electrons in the SmFeO_3_ system, since such an interaction coexists and lasts even up to room temperature. It is the strong Sm-4f and Fe-3d electrons interaction that makes SmFeO_3_ single crystal unique and may be put in use for designing novel spin switching devices in the near future. For device technology where small magnetic field of mT is applied by current pulses, we might propose substitutional doping Sm by other rare earth, e.g. Nd, to further lower the switching field at room temperature.

In conclusion, we have studied the temperature-induced multiple magnetic transition properties of single-crystal SmFeO_3_, by demonstrating spin reorientation, compensation and switching phenomena that originated from the Sm-4f and Fe-3d electrons and their interaction. In particular, SmFeO_3_ possesses an extremely useful magnetization that is very sensitive to small field perturbations even at room temperature. This makes SmFeO_3_ an easily manipulable candidate for practical application in spin switching devices by using very low field. Furthermore, this study indicates that the significant role of rare earth Sm ions in RFeO_3_ or other perovskite ABO_3_ compounds should be focused both in experimental and theoretical study. Sm-4f electrons may trigger more novel effects of spin state transitions^25^ near room temperature, which is desirable for the use of spin in future spintronics.

## Methods

Single crystal of SmFeO_3_ was grown in a four-mirror optical-floating-zone furnace (FZ-T-10000-H-VI-P-SH, Crystal Systems Corp.) using four 1.5 kW halogen lamps as the infrared radiation source with flowing air. The temperature of the molten zone was precisely controlled by adjusting the power of the lamps. During the growth process, the molten zone moved upwards at a rate of 3 mm h^−1^, with the seed rod (lower shaft) and the feed rod (upper shaft) counter rotating at 30 rpm in air flow of 5 L min^−1^. The compositional homogeneity and crystal morphology were analyzed by X-ray diffraction (XRD), and scanning electron microscopy (SEM) with energy-dispersive X-ray spectroscopy (EDX). All results confirmed the high homogeneity of the crystals studied.

Measurements of magnetization as a function of temperature (*M-T*) and magnetic field (*M-H*) were performed using the Quantum Design Physical Property Measurement System (type PPMS-9) for temperatures below 400 K and the Lakeshore Vibrating Sample Magnetometer (VSM, type 7407) for temperatures from 300 K to 750 K. For the zero-field-cooling (ZFC) measurement, the sample was progressively cooled down under zero magnetic field until the temperature of 3 K is achieved. A magnetic field was then applied, and the sample was heated under this field to measure the magnetization as the temperature increases. For the field-cooling (FC) measurement, the sample was progressively cooled down to the temperature of 3 K under an applied magnetic field. Then, the sample was heated under the same field to measure the magnetization as the temperature increases.

## Author Contributions

**Figure 1:**
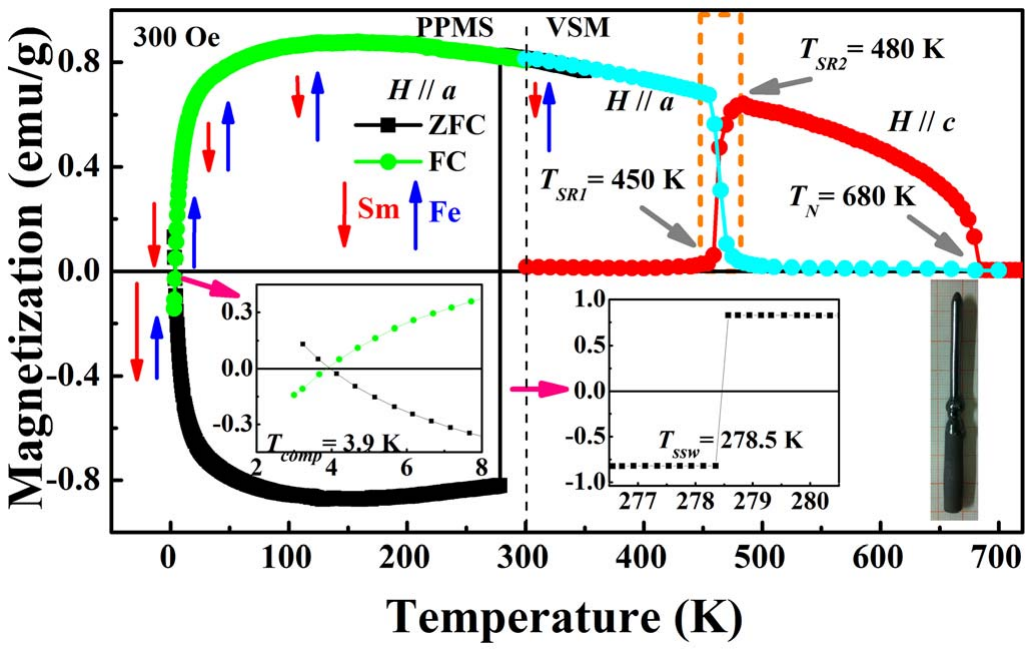
(Left part below 350 K) Temperature dependence of the magnetizations of SmFeO_3_ single crystal measured along the *a*-axis under ZFC (black squares) and FC (green circles) regimes. The measuring fields are *H* = 300 Oe, and the measurements are performed in a process of increasing the temperature. The left inset shows zoom-in region near the critical temperature, and the right inset shows zoom-in region near the magnetic jump at *T_ssw_* = 278.5 K. The red and blue arrows schematize the evolution of the magnetizations arising from Sm (in red) and Fe (in blue) ions for the FC curve (green circles). (Right part above 300 K) Temperature dependence of the magnetizations of SmFeO_3_ single crystal measured along the *a*- and *c*-axes (cyan and red circles). The dashed rectangle highlights the spin reorientation transition from *a*-axis to *c*-axis for the FM vector of Fe-sublattice between 450 and 480 K. The photograph at lower right corner illustrates single crystal SmFeO_3_ grown by the floating-zone method.

**Figure 2:**
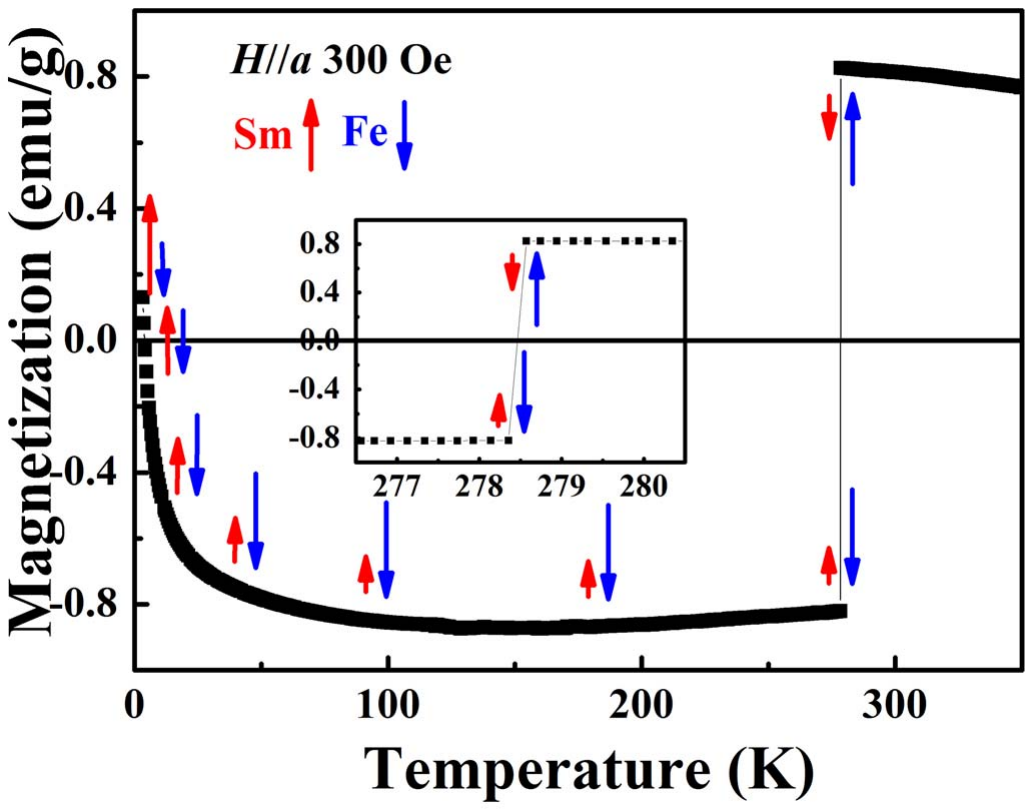
Temperature dependence of the magnetization of SmFeO_3_ single crystal measured along *a*-axis under ZFC regime. The measuring field is 300 Oe, and the measurements are performed by increasing the temperature. The arrows schematize the predicted evolution of the magnetizations arising from Sm (in red) and Fe (in blue) ions. The inset shows zoom-in region near the critical temperature of spin switching transition.

**Figure 3:**
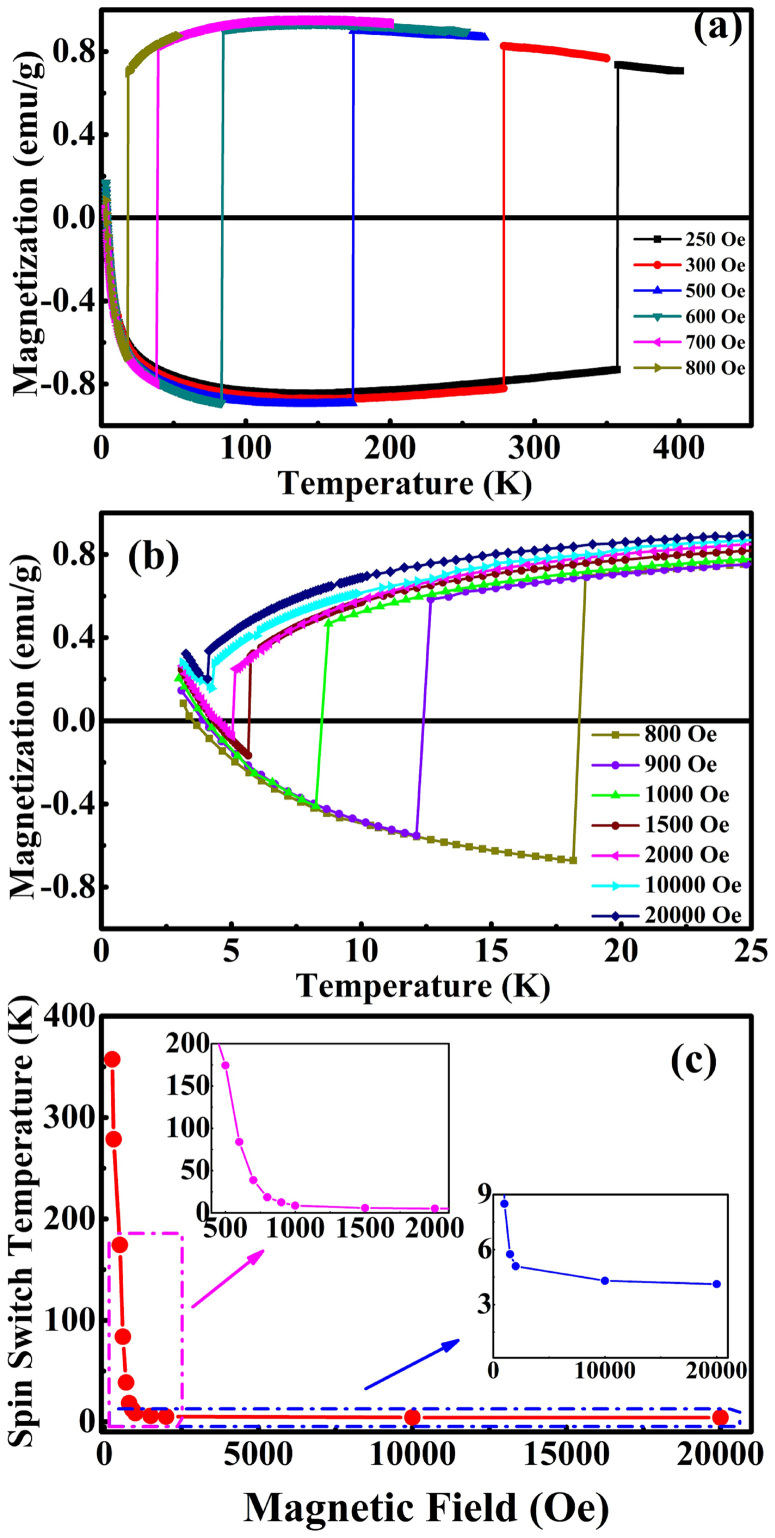
Temperature dependence of the magnetization of SmFeO_3_ single crystal under different applied fields, measured along *a*-axis in ZFC regime. (a), the field *H* changes from 250 Oe to 800 Oe; (b), the field *H* changes from 800 Oe to 20000 Oe; (c), the relationship of spin switching temperature *T*_ssw_ versus magnetic field *H*.

**Figure 4:**
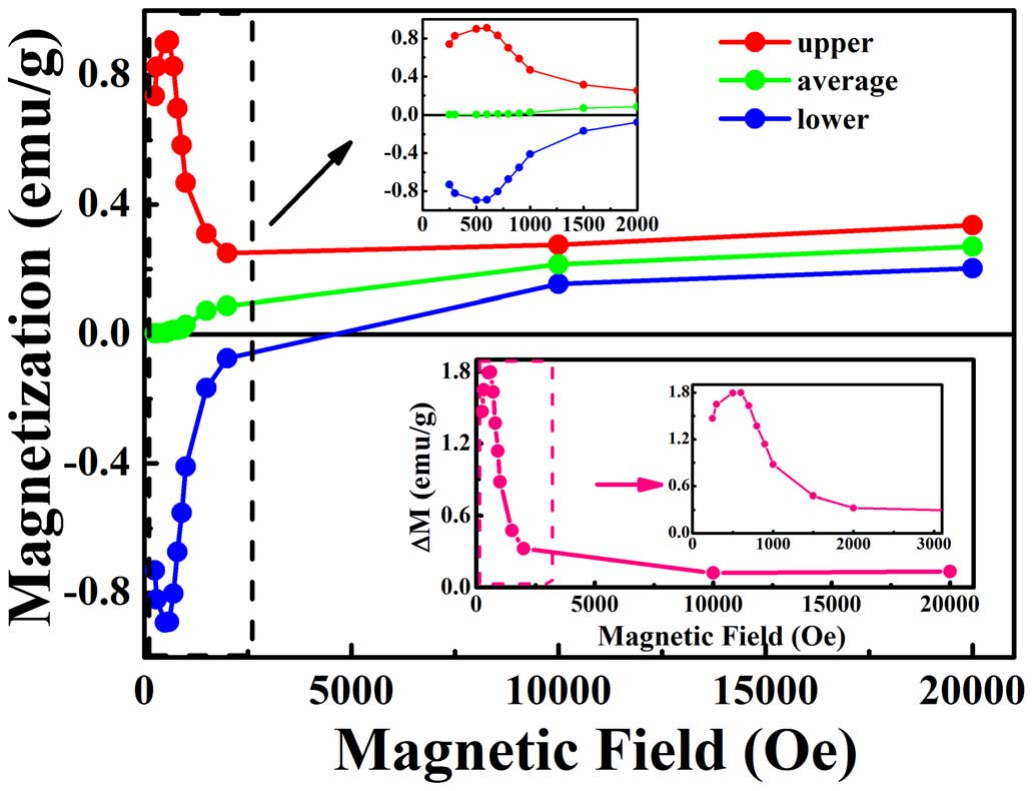
Magnetic field dependence of the magnetization at the spin switching temperature of SmFeO_3_ single crystal, where the “lower” and “upper” refer to the magnitudes of magnetization before and after the spin switching transition, respectively; the “average” refers to the midpoint magnetization of the “lower” and “upper”; the upper inset shows zoom-in region marked by the dashed black rectangle for the field *H*<2000 Oe; the lower inset shows the magnetization jump (Δ*M*) before and after the spin switching transition as a function of magnetic field.

**Figure 5:**
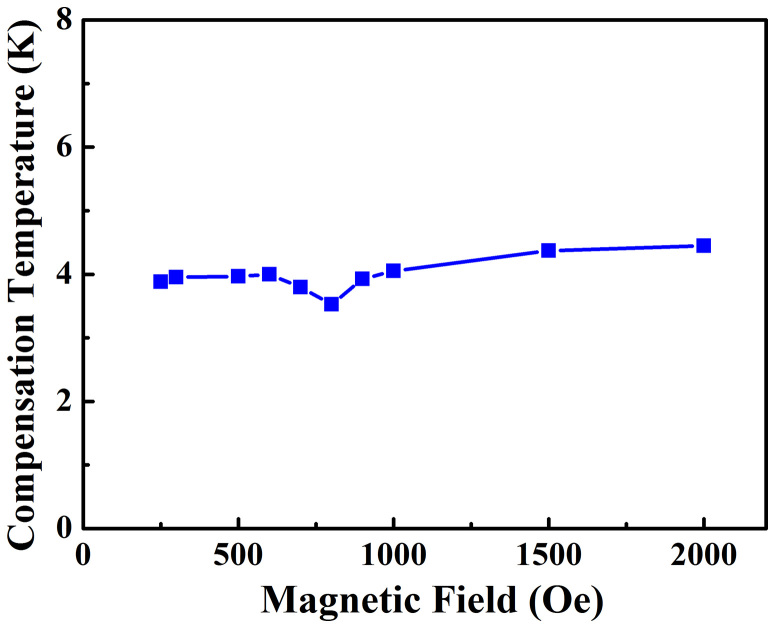
Magnetic field dependence of compensation temperature of SmFeO_3_ single crystal.

S.C., H.Z. and W.R. conceived the idea for this project and wrote the manuscript. S.C., H.Z. and B.K. did all the experiments and prepared the figures 1–5. S.C., H.Z., J.Z. and W.R. reviewed the manuscript. All authors contributed to the discussion of the results and commented on the manuscript.

## References

[b1] Kimel A. V. *et al.* Inertia-driven spin switching in antiferromagnets. Nat. Phys. 5, 727 (2009).

[b2] Ehresmann A. *et al.* Asymmetric Magnetization Reversal of Stripe-Patterned Exchange Bias Layer Systems for Controlled Magnetic Particle Transport. Adv. Mater. 23, 5568 (2011). 22052724 10.1002/adma.201103264

[b3] Yusuf S. M., Kumar A. & Yakhmi J. V. Temperature- and magnetic-field-controlled magnetic pole reversal in a molecular magnetic compound. Appl. Phys. Lett. 95, 182506 (2009).

[b4] Kimel A. V. *et al.* Ultrafast non-thermal control of magnetization by instantaneous photomagnetic pulses. Nature 435, 655 (2005). 15917826 10.1038/nature03564

[b5] Yuan S. J. *et al.* Spin switching and magnetization reversal in single-crystal NdFeO_3_. Phys. Rev. B 87, 184405 (2013).

[b6] Kimel A. V., Kirilyuk A., Tsvetkov A., Pisarev R. V. & Rasing T. Laser-induced ultrafast spin reorientation in the antiferromagnet TmFeO_3_. Nature 429, 850 (2004). 15215858 10.1038/nature02659

[b7] Jeong Y. K., Lee J. H., Ahn S. J. & Jang H. M. Temperature-induced magnetization reversal and ultra-fast magnetic switch at low field in SmFeO_3_. Solid State Commun. 152, 1112 (2012).

[b8] White R. M., Nemanich R. J. & Herring C. Light scattering from magnetic excitations in orthoferrites. Phys. Rev. B 25, 1822 (1982).

[b9] Tsymbal L. T. *et al.* Magnetic and structural properties of spin-reorientation transitions in orthoferrites. J. Appl. Phys. 101, 123919 (2007).

[b10] Chen L. *et al.* The role of 4f-electron on spin reorientation transition of NdFeO_3_: A first principle study. J. Appl. Phys. 111, 103905 (2012).

[b11] de Jong J. A., Kimel A. V., Pisarev R. V., Kirilyuk A. & Rasing T. Laser-induced ultrafast spin dynamics in ErFeO_3_. Phys. Rev. B 84, 104421 (2011).

[b12] Bossini D. *et al.* Time-resolved nonlinear infrared spectroscopy of samarium ions in SmFeO_3_. Phys. Rev. B 87, 085101 (2013).

[b13] Wang K. F., Liu J. M. & Ren Z. F. Multiferroicity: the coupling between magnetic and polarization orders. Adv. Phys. 58, 321 (2009).

[b14] Lee J. H. *et al.* Spin-Canting-Induced Improper Ferroelectricity and Spontaneous Magnetization Reversal in SmFeO_3_. Phys. Rev. Lett. 107, 117201 (2011). 22026697 10.1103/PhysRevLett.107.117201

[b15] Tokunaga Y., Iguchi S., Arima T. & Tokura Y. Magnetic-Field-Induced Ferroelectric State in DyFeO_3_. Phys. Rev. Lett. 101, 097205 (2008). 18851654 10.1103/PhysRevLett.101.097205

[b16] Belov K. P., Kadomtseva A. M., Ledneva T. M., Ovchinnikova T. L. & Timoreeva V. A. Features of the Temperature Dependence of the Magnetization of Thulium Orthoferrite. JETP Lett. 2, 161 (1965).

[b17] Bozorth R. M., Kramer V. & Remeika J. P. Magnetization in Single Crystals of Some Rare-Earth Orthoferrites. Phys. Rev. Lett. 1, 3 (1958).

[b18] White R. L. Review of Recent Work on the Magnetic and Spectroscopic Properties of the RareEarth Orthoferrites. J. Appl. Phys. 40, 3 (1969).

[b19] Zhao H. Z. *et al.* Enhanced 4f-3d interaction by Ti-doping on the magnetic properties of perovskite SmFe_1−x_Ti_x_O_3_. J. Appl. Phys. 114, 113907 (2013).

[b20] Marshall L. G. *et al.* Magnetic coupling between Sm^3+^ and the canted spin in an antiferromagnetic SmFeO_3_ single crystal. Phys. Rev. B 86, 064417 (2012).

[b21] Derkachenko V. N., Kadomtseva A. M., Timofeeva V. A. & Khokhlov V. A. Temperature hysteresis of the magnetization in orthoferrites at the compensation point. JETP Lett. 20, 104 (1974).

[b22] Jung J. S. *et al.* Magnetocapacitive effects in the Néel N-type ferrimagnet SmMnO_3_. Phys. Rev. B 82, 212403 (2010).

[b23] Cheng J. G. *et al.* Exchange field on the rare earth Sm^3+^ in a single crystal perovskite SmMnO_3_. Phys. Rev. B 84, 104415 (2011).

[b24] Hong F., Cheng Z. & Wang X. Strong 4f electron interaction and magnetic ordering modification in Nd_1-x_Er_x_MnO_3_ (0 ≤ x ≤ 0.5). Appl. Phys. Lett. 99, 192503 (2011).

[b25] Ren Y. *et al.* Temperature-induced magnetization reversal in a YVO_3_ single crystal. Nature 396, 441 (1998).

